# Findings in Danish long-term care facilities in the first year of the SARS-CoV-2 pandemic

**DOI:** 10.1007/s41999-023-00793-y

**Published:** 2023-05-18

**Authors:** Laura Espenhain, Tjede Funk, Asja Kunøe, Manon Chaine, Karina Lauenborg Møller, Brian Kristensen

**Affiliations:** 1 Department of Infectious Disease Epidemiology and Prevention, Statens Serum Institut, Copenhagen, Denmark; 2 The European Programme for Intervention Epidemiology Training (EPIET) Fellowship, European Centre for Disease Prevention and Control, (ECDC), Solna, Sweden; 3 Division of Infectious Disease Preparedness, Statens Serum Institut, Copenhagen, Denmark

**Keywords:** SARS-CoV-2, COVID-19, Outbreaks, Long-term care facilities, Denmark, Automated surveillance

## Abstract

**Purpose:**

To describe the occurrence of SARS-CoV-2 infections, deaths and outbreaks among residents in Danish long-term care facilities (LTCFs) from February 2020 to February 2021.

**Methods:**

Danish COVID-19 national register data from a newly implemented automated surveillance system was used to describe incidence rate and deaths (per 1000 residents’ years), number of tests, SARS-CoV-2 infections and outbreaks among LTCF residents. A case was defined as a LTCF resident with a positive SARS-CoV-2 PCR test. An outbreak was defined as two or more cases in one LTCF within a 14-day period, and considered closed if no new cases had occurred within 28 days. Death was defined as occurring within 30-days of a positive test.

**Results:**

A total of 55,359 residents living in 948 LTCFs were included. The median age of the residents was 85 years and 63% were female. There was a total of 3712 cases found among residents across 43% of all LTCFs. Nearly all (94%) cases were linked to outbreaks. Higher numbers of cases and outbreaks were seen in Denmark’s Capital Region compared to other regions. Overall, 22 SARS-CoV-2 deaths and 359 deaths (non-SARS-CoV-2) per 1000 resident years were identified in the study period.

**Conclusion:**

Less than half of LTCFs identified any cases. The majority of cases were linked to outbreaks, emphasizing the importance of preventing introductions of SARS-CoV-2 into the facilities. Furthermore, it highlights the need to invest efforts into infrastructures, routine procedures and monitoring of SARS-CoV-2 in LTCFs to limit the introduction and the spread of SARS-CoV-2.

## Key summary points

**Aim:**

By use of a newly established national automated registry-based surveillance system, this study describes SARS-CoV-2 infections, deaths and outbreaks among Danish long-term care facility residents in the first year of the COVID-19 pandemic 2020/2021. Also, to assess the magnitude of SARS-CoV-2 transmission in these settings

**Findings:**

Less than half of the Danish long-term care facilities identified at least one case. Almost all (94%) cases were linked to an outbreak. Regional differences were observed with the Capital Region having substantial higher numbers of both cases and outbreaks compared to the other four Danish regions.

**Message:**

A national automated registry-based surveillance system is key in monitoring and analysing patterns in numbers and outbreaks of SARS-CoV-2 in long-term care facilities. The majority of SARS-CoV-2 cases among long-term care facility residents were linked to an outbreak, highlighting the importance of investing efforts into avoiding introductions of the virus.

## Introduction

Across Europe, in May 2020, the coronavirus disease (COVID-19) deaths among long-term care facility (LTCF) residents accounted for a large proportion of all COVID-19 deaths [1, 2]. LTCF residents are generally vulnerable and often have multiple comorbidities. Increased age, impaired cognitive and physical function in people with COVID-19 are independent risk factors for all-cause 30-day mortality [3]. The combination of increased age and high prevalence of diseases such as diabetes, cancer, chronic heart or respiratory diseases, all significantly contribute to LTCF residents being at higher risk of severe outcomes of COVID-19 [2, 4, 5]. Consequently, COVID-19 has caused significant morbidity and mortality in LTCFs [1, 6–8]. To protect the older population, there is a need to detect, prevent and control COVID-19 outbreaks in LTCFs as well as continuously monitor the situation [1, 9–11].

In Denmark, the average age to move into a LTCF was 83.7 years and 50% of the LTCF residents had one or more chronic diseases [12, 13]. Early during the pandemic, the Danish government implemented different protective measures in LTCFs. These protective measures included: visitor restrictions (a formal ban was introduced April 4, 2020 allowing only visits in critical situations), increased testing among residents and staff (in the case of one positive test from staff or resident) as well as increased infection control measures for instance in the form of using face masks and other personal protective equipment [14–16]. The LTCF population was one of the first target groups for the Danish COVID-19 vaccination programme which was rolled out in December 2020 [17]. Prior to the COVID-19 pandemic, there was no national surveillance system to follow the occurrences and development of outbreaks of infectious diseases and deaths in LTCFs in Denmark. A register-based automated surveillance system was therefore set up and implemented in 2020 to monitor infections with SARS-CoV-2 in Danish LTCFs [18]. It was used as an information source and basis in the discussions on implementation of precaution measures or changing of testing strategies in these settings.

The aim of this study was to describe SARS-CoV-2 infections, outbreaks and deaths among LTCF residents in Denmark using the Danish national surveillance data from LTCFs during the first year of the pandemic and to assess the COVID-19 situation in LTCFs during the initial period when vaccines were not available.

## Methods

### Study population

This was a descriptive registry-based study of SARS-CoV-2 infections in LTCF residents during the first year of the pandemic. The study population included residents with a registered address in a Danish LTCF during the period of February 23, 2020 to February 28, 2021. February 23, 2020 was the date of the first SARS-CoV-2 real-time reverse transcription polymerase chain reaction (PCR) test taken in a LTCF resident.

### Data sources

The National Danish Microbiology Database (MiBa) formed the basis of the Danish COVID-19 surveillance system. MiBa automatically receives all microbiological test results from all Departments of Clinical Microbiology in Denmark as well data from TestCenter Denmark [9, 19]. Through a national identification number (CPR number), a unique identifier given at birth or upon immigration, information from a number of other national registries (data used here from civil registry, LTCF-registry, cause of death registry) was linked to the SARS-CoV-2 test information on a daily basis [20–22]. Information on SARS-CoV-2 tests (e.g. sample date and test result) was extracted only for persons with a registered address at a LTCF. Additionally, data was linked to information on moving in and out dates of the facility as well as the date of death when it occurred. The LTCF-registry includes both public and private institutions for older persons who require support and cannot live independently. All LTCFs had to follow public law, but we had no access to detailed information about the distribution of public versus private institutions [22]. The linkage of different data sources thus allowed the automatic retrieval of information and did not rely on manual data reporting of positive tests among residents by LTCFs or others.

### Test and vaccination programme strategies in the study period

In Denmark, indications for testing of SARS-CoV-2 are regulated by the Danish Health Authority (DHA) and supported by a continuous dialogue between the National Patient Safety Authority and the municipalities. The testing strategies changed a number of times during the study period besides a shift mid-March from confinement to mitigation strategy. The DHA issued recommendations for testing to be applied nationally at LTCFs and the recommendations were uniform across centres.

All measures were recommendations and occurred in the following timeline: From the end of March 2020: PCR-testing of residents if symptoms were suspected. From end of April 2020: PCR-testing of close contacts (residents and staff) after the occurrence of a case. From the beginning of May 2020: PCR-test of asymptomatic residents in certain situations. From the beginning of June 2020: Regular PCR-testing of staff (once every two weeks) in places with high community transmission. From end of November 2020: Regular PCR-testing of staff. From January 2021: PCR-test among staff one to two times weekly.

The Danish COVID-19 vaccination programme started December 27, 2020. Residents at the LTCFs were the first target group.

### Definitions

A case was defined as a resident with a positive SARS-CoV-2 PCR test. We included only the first positive test for each LTCF resident, meaning that reinfections (in the Danish surveillance defined as a positive PCR test occurring > 60 days after the first positive test) were excluded from this study. Since a very low number of reinfections (0.75% (*n* = 28) of the 3712 residents who tested positive) was identified in our study population during the study period we did not consider it to have any significant relevance to our results or estimations of case-fatality rate.

In instances where multiple PCR tests had been carried out in one person in one day, we only included one, the positive if applicable.

A SARS-CoV-2 death was defined as a death occurring within 30 days after the first positive PCR test. We calculated the case-fatality rate by dividing the number of SARS-CoV-2 deaths by the number of cases.

A SARS-CoV-2 outbreak at a LTCF was defined as two or more cases in one LTCF within a 14-day period. It is based on the definition suggested by the United Kingdom Health Security Agency [23]. An outbreak was defined to have ended when no new case(s) were observed 28 days after the last confirmed case. The length of an outbreak was defined as the period between the sample date of the first and the last case. The term introductory case was used to describe the first case in an outbreak or the first observed case within a 15-day period in a LTCF without an ongoing outbreak. When only one case in a LTCF was registered within a 14-day period, this was termed a sporadic case. An index case was defined as the first resident who tested positive in an outbreak at a LTCF.

### Analyses

We calculated time at risk of infection and death and expressed it as resident years. We defined the start of time at risk from February 23, 2020 or from move-in date to the LTCF, whichever came last. The end of time at risk was defined as move-out date from the LTCF, date of death or end of study period (February 28, 2021) depending on which came first. As for the calculation of risk of infection, residents were censored from the date of the first positive test in the analyses concerning infections.

Incidence rates per 1000 resident years were calculated by dividing the number of cases, SARS-CoV-2 deaths, all-cause deaths and PCR tests by the number of resident years accumulated during the study period. We calculated the time from the sample date of an index case to 50% of the residents in the LTCF had been tested.

Using negative binomial regression, LTCFs with at least one case during the study period were compared with LTCFs without cases in terms of all-cause mortality excluding SARS-CoV-2 deaths. This analysis was adjusted for size of the LTCF, proportion of women, average age of the residents and region.

The data preparation and registry linkage were done using SAS version 9.4. All analyses were performed in Stata version 14.2 and visualisations were performed in Microsoft Excel.

## Results

### Characteristics of the study population

During the study period, Denmark had a total of 948 LTCFs spread across the country, eight of which had been closed or merged with other LTCF by February 28, 2021. The median size was 38 residents (interquartile range (IQR): 24–55 residents) and the LTCFs were larger in size in the Capital Region (median 58 residents) compared to the other four regions (median 31–38 residents). In total, 55,359 persons were registered with address at a LTCF and altogether 41,360 resident-years accumulated during the study period (Tables 1 and 2). The median age of a resident through the study period was 85 years (IQR 78–91 years). Among all the residents 14,847 (27%) died during the study period, equivalent to 359 per 1000 resident years (Table 1).

**Table 1:** Overall and by region: General population and cumulated incidence during the study period. Long-term care facilities: number and size. Residents at long-term care facilities: Number of residents, median age and proportion female residents, number of all-cause deaths during the study period and number of SARS-CoV-2 tests per residents during the study period, from February 23, 2020 to February 28, 2021, Denmark

	Capital	Zealand	Southern Denmark	Central Denmark	North Denmark	Total
Population by January 1st, 2021	1,855,084	838,840	1,223,634	1,332,048	590,439	5,840,045
Proportion of the population who tested positive during the study period (%)	5.41	3.51	2.33	2.79	2.45	3.59
Number of LTCF*	193	141	225	253	136	948
Size of LTCF* (number of residents per end of study period), median [IQR**]	58 [41–83]	38 [23–50.5]	35.5 [25–48.5]	31 [20–49]	32.5 [23–45]	38 [24–54.5]
Number of LTCF* residents registered during study period	16,824	7664	11,857	12,407	6607	55,359
Age (median [IQR**]) of LTCF* residents	85 [78–91]	84 [77–90]	85 [78–90]	85 [78–90]	86 [79–91]	85 [78–91]
Females (%)	65	63	61	62	62	63
Number of all-cause deaths in LTCF residents during the study period	4593	2084	3110	3303	1757	14,847
Number of SARS-CoV-2 PCR tests per resident during the study period	6.2	3.0	2.2	2.3	2.6	3.6

*LTCF: Long-term care facilities *IQR: inter-quartile range

**Table 2:** SARS-CoV-2 cases, SARS-CoV-2 deaths, and case-fatality rate among long-term care facility residents in total and per region, from February 23, 2020 to February 28, 2020, Denmark

Region	SARS-CoV-2 cases			SARS-CoV-2 deaths			Case-fatality **
	Resident years for risk of infection*	Total number of reported SARS-CoV-2 cases	SARS-CoV-2 cases per 1,000 resident years	Resident years for risk of death*	Total number of SARS-CoV-2 deaths	SARS-CoV-2 deaths per 1,000 resident years	
Capital	11,998	2173	181	12,470	534	43	25
Zealand	5576	440	79	5676	96	17	22
Southern Denmark	8872	263	30	8913	72	8	28
Central Denmark	9227	599	65	9326	153	16	26
North Denmark	4934	237	48	4975	72	14	30
Total	40,607	3712	91	41,360	927	22	25

*Time at risk was expressed in resident years. Defined as the start of time at risk from February 23, 2020 or from move-in date to the LTCF, whichever came last. The end of time at risk was defined as move-out date from the LTCF, date of death or end of study period (February 28, 2021) depending on which date came first. For the calculation of risk of infection, residents were censored from the date of the first positive test in the analyses concerning infections.

**Case-fatality calculated by dividing the number of SARS-CoV-2 deaths by the number of SARS-CoV-2 cases

### SARS-CoV-2 cases and deaths among LTCF residents

**Fig. 1:**
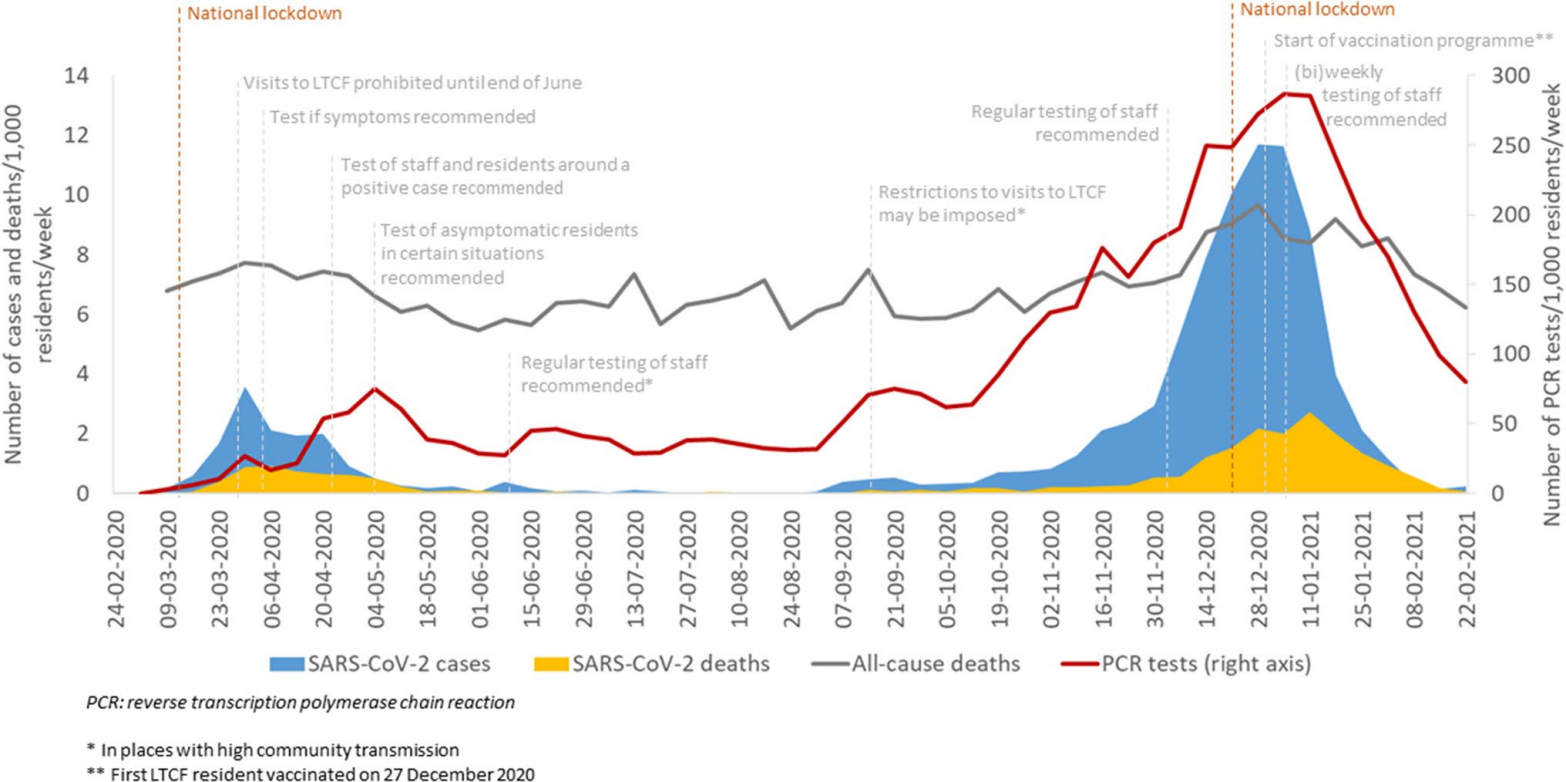
Number of SARS-CoV-2 cases, SARS-CoV-2 deaths, all-cause deaths and performed PCR-tests among LTCF residents per 1000 residents per week and major restrictions and recommendations in LTCFs, February 23, 2020 to February 28, 2021, Denmark

On March 12, 2020, the first resident on a LTCF tested positive for SARS-CoV-2. During the study period, 3,712 (7%) LTCF residents tested positive for SARS-CoV-2 and 927 died within 30 days. It corresponded to 91 cases per 1000 resident years, and 22 SARS-CoV-2 deaths per 1000 resident years and a case-fatality rate of 25% (Table 2). Altogether, we identified 587 introductory cases of which 239 (41%) were sporadic cases and 348 (59%) were linked to outbreaks (Table 3). Between March and early May 2020, the number of cases deaths increased followed by a second and even larger peak between December and January 2020/2021 (Fig. 1).

**Table 3:** Number of long-term care facilities with residents with SARS-CoV-2 infection, outbreaks and characteristics of reported outbreaks by region, from February 23, 2020 to February 28, 2021, Denmark

Region	Long-term care facility				Outbreaks at long-term care facilities			
	LTCF with ≥ 1 confirmed SARS-CoV-2 case		LTCF with ≥ 1 reported outbreak		Outbreaks	Cases involved in outbreak	Size of outbreak in cases	Length of outbreak in days
	n	%	n	%	n	n	Median [IQR*], maximum	Median [IQR*], maximum
Capital	165	85	141	73	179	2056	9 [3–16], 68	17 [8–31], 77
Zealand	65	46	48	34	52	405	5 [3–9.5], 34	14 [9–20] , 57
Southern Denmark	50	22	36	16	36	243	5 [3–8.5], 37	9 [5.5–18], 40
Central Denmark	84	34	48	19	54	554	8 [4–13], 49	15.5 [1023], 57
North Denmark	41	31	25	19	27	215	5 [2–12][, 34	12 [4–17], 36
Total	405	43	298	32	348	3473	7 [3–13], 68	15 [8–25], 77

LTCF: Long-term care facility

*IQR: Inter-quartile rang

During the study period, 43% (*n* = 405) of LTCFs identified at least one case, with large regional differences (Table 3). Most cases and deaths were identified in the Capital Region with 85% of the LTCFs having cases among their residents (Table 3). As a consequence, the number of cases and deaths per 1000 resident years were two to six times higher, respectively 181 and 43, in the Capital Region compared to the other regions (Table 2).

In the LTCFs which had at least one confirmed case of SARS-CoV-2 during the study period (n = 405), we observed no difference in the number of all-cause mortality, this is excluding SARS-CoV-2 deaths compared to LTCFs (n = 543) that did not have any confirmed cases of SARS-CoV-2 during the study period (IRR = 1.046 (95% CI 0.997 – 1.097, *p*-value = 0.06), when taking region, number of residents, proportion of women and average age of the residents, into account.

### SARS-CoV-2 tests among LTCF residents

According to the different recommendations for testing (Fig. 1) the following numbers of tests per week per 1,000 residents were performed: From March 30, 2020 to April 30, 2020: 38 tests. From May 1, 2020 to June 1, 2020: 52 tests. From June 2, 2020 to November 30, 2020: 68 tests. From December 1, 2020 to January 1, 2021: 237 tests. From January 2, 2021 to February 28, 2021: 199 tests.

From mid-October 2020, the number of PCR-tests performed increased markedly from around 64 tests per 1000 residents per week to 274 per 1000 per week at the end of December 2020 (Fig. 1). In contrast, the percentage of residents testing positive decreased from 16% to below 5% during this period. Regional test activity followed the overall trend, but ranged from 2.2 tests (Region Southern Denmark) to 6.2 tests (Capital Region) per resident during the study period (Table 1). Following the identification of an index case, 2 days (median) passed until 50% of the residents at the LTCF had been tested.

### Outbreaks in LTCF

A total of 348 SARS-CoV-2 outbreaks with 3,473 cases in 298 (32%) LTCFs were identified (Table 3). Cases being a part of an outbreak accounted for 93.5% of all SARS-CoV-2 cases among LTCF residents.

Outbreaks ranged in size between two and 68 cases, with a median of seven cases (IQR 3–13). During the study period, 18% (n = 61) of outbreaks involved two cases and 5% of the outbreaks (n = 17) involved 30 cases or more. The majority of outbreaks (64%, n = 226) involved 10 or fewer cases.

Most outbreaks were identified in the Capital Region (n = 179; 51%), followed by the Region of Central Denmark and Zealand (respectively 54 and 52, Table 3). The median number of cases that were part of an outbreak as well as the length of outbreaks were the largest in the Capital and Central Region of Denmark (Table 3).

In total, 46 of the LTCFs (5%) experienced more than one outbreak during the study period. The vast majority of these LTCFs (n = 34; 74%) were located in the Capital Region.

## Discussion

Based on national register-based surveillance data, this study provides a thorough overview of SARS-CoV-2 cases, outbreaks, and deaths among LTCF residents in Denmark. We observed that 93.5% of all cases amongst LTCF residents and 59% of introductory cases were linked to outbreaks. A total of 3712 SARS-CoV-2 cases (91/1000 resident years) were detected. Less than half of LTCFs had SARS-CoV-2 cases (43% of LTCFs) and outbreaks (32% of LTCFs) among its residents. Marked regional differences were observed in LTCFs, with the Capital Region tending to be more affected. The study period covered the first year of the pandemic and briefly overlapped with the start of the COVID-19 vaccine roll-out. Consequently, the study population was SARS-CoV-2 naïve almost the entire period. However, by the end of the study period February 28, 2021, the majority of the LTCF residents had been vaccinated and SARS-CoV-2 cases declined [24].

The number of detected cases and SARS-CoV-2 deaths among residents of LTCFs was lower in the beginning of the pandemic and increased markedly during the winter of 2020/2021. This trend followed patterns seen in LTCF staff as well as in the general population of Denmark and other countries, but also coincided with an increased testing activity [24, 25]. Therefore, trends need to be interpreted with caution. Nationwide seroprevalence studies in the general Danish population, carried out in May, August and December 2020, showed that SARS-CoV-2 was indeed not widespread in spring 2020, supporting that the trend observed was not merely seen due to changes in testing strategies [26].

A Danish study from 2019, investigating mortality and morbidity in Danish nursing homes found 70% of residents still alive one year after nursing home admission [13]. This result is lower, but within the same range as the one-year mortality of all-cause death in our study, and even more so when excluding deaths related to COVID-19. Studies from other countries carried out before the pandemic in general support that LTCF residents are a vulnerable population, but also that the population group may differ across countries, previous studies have reported one-year mortality rates ranging from 17.4 in France to 31.8% in Norway and 35% in the US [27–29] This stresses the importance of investing efforts into avoiding introductions of infectious diseases into this already vulnerable population.

Compared to the great regional differences observed in Denmark, a study from England including both residents and staff found that more than two-thirds of the LTCFs identified cases and around half experienced outbreaks [25]. Various factors may have contributed to the finding that the number of cases and proportion of affected LTCFs were two to six times higher in the Capital Region compared to other regions. Firstly, the Capital Region had double the cumulative community incidence compared to the rest of Denmark which may have increased the risk of SARS-CoV-2 introductions through staff, visitors or when residents moved into the facility or were discharged from the hospital. This risk factor is also reported by others [30–35]. Secondly, more tests were conducted in the Capital Region compared to other regions. Whether this was a result of screening due to contact tracing, or increased attention remains unknown, but may nevertheless have led to the identification of more cases in the Capital Region. The case facility rate observed in the three regions towards the west of Denmark seemed to be higher. We do not have an explanation for this result, but the higher testing rates in the Capital and Zealand regions could have affected the case facility rate. Finally, LTCFs in the Capital Region tended to be larger with more residents per LTCF compared to the other regions. This could have resulted in more densely populated LTCFs, more staff and visitors, and more mixing and contact between individuals, which could make it easier for an infection to spread once introduced. One systematic review found higher numbers of staff members to be associated with a higher probability of outbreaks [30]. However, we do not have data to shed light on this possible relationship.

A Danish report from autumn 2020 showed that municipalities prioritising hygiene efforts in an organised way had a lower cumulative SARS-CoV-2 incidence among residents at LTCFs compared to LTCFs in municipalities which were not organised [36]. However, the extent to which implemented hygiene measures could explain this trend is not known in the current study. One study investigated nursing home quality ratings by health inspections (including quality measures related to infection control) and found lower COVID-19 rates among residents in nursing homes with the highest ratings [37]. Two systematic reviews did not find consistent associations between COVID-19 lower rates and implemented non-pharmacological measures or quality ratings including infection control measures [30, 38].

Our data indicated that most cases and more than half of the introductory cases in LTCFs were linked to outbreaks. This emphasises the importance of investing specific efforts into avoiding such introductions as well as the need to have the infrastructure and routine procedures in place for early detection and reaction, in order to limit spread when introductions occur. A Danish study in one residential care home with 114 beds in the Capital Region of Denmark found high risk of SARS-CoV-2 transmission (26% of staff and 50% of residents) once the virus was introduced [39]. As a consequence of testing residents and staff from April 2020 in Denmark, the probability of an increase in identified infected residents was likely and might partly explain the high percentage of cases linked to outbreaks.

Before the COVID-19 pandemic, no routine surveillance systems covering LTCFs existed in Denmark. The system was set up, linking different registers, to monitor the development of SARS-CoV-2 cases among LTCF residents, a particularly vulnerable population group. It is a major strength of the study that the reported LTCF surveillance data were national. The surveillance system included all LTCFs and test activity was relatively high, thus the findings are considered representative for this setting in Denmark. The data derived through this surveillance system proved to be a useful information source and basis when discussing implementation of precaution measures or changing of testing strategies in these settings. Aggregated data has been publicly available online on a weekly basis since spring 2020 and detailed reports were shared with relevant authorities regularly [18].

Our study was limited on the outbreak definition, where it was assumed that infections clustering in time and place were linked. It did not take into consideration the SARS-CoV-2 variant or the genetic relatedness of cases. It is therefore possible that multiple introductions were misclassified as one outbreak, resulting in a potential overestimation of the number of outbreaks. Another limitation was that we were not able to link staff to a specific LTCF. This would have allowed us to better explore and capture SARS-CoV-2 introduction and circulation of SARS-CoV-2 in LTCFs. Consequently, the overall number of introductions might have been underestimated in this study. A third limitation was the introduction of the recommendation of antigen testing for the staff at the LTCFs, from mid-December 2020. Antigen tests were not a part of the national SARS-CoV-2 surveillance at the time of the study. Lastly, the diagnostic test strategy used has conditioned the results shown in the study, as well as the definition of “death related to or not” to SARS-CoV-2.

In conclusion, during the first year of the COVID-19 pandemic, SARS-CoV-2 cases and outbreaks were identified in residents in less than half of the Danish LTCFs. More than half of the introductory cases and the majority of cases in total were linked to outbreaks emphasizing the importance of investing efforts into avoiding introductions of SARS-CoV-2. The Danish automated national COVID-19 surveillance system was set up to include LTCFs. When introductions occurred, it was shown to be pivotal to have the infrastructure and routine procedures in place for early detection and reaction to limit spread of SARS-CoV-2.

## Data Availability

De-identified data are available for access to members of the scientific community for non-commercial use. More information, including on how to apply, is available through Forskerservice at The Danish Health Data Authority. Applications will be reviewed on the basis of relevance and scientific merit.
